# A Feverish Liver Transplanted Child

**Published:** 2011-02-01

**Authors:** B. Geramizadeh

**Affiliations:** *Department of Pathology, Transplant Research Center, Shiraz University of Medical Sciences, Shiraz, Iran*

A 5-year-old boy underwent orthotopic liver transplantation (OLT) for cirrhosis secondary to tyrosinemia. His immediate post-transplant course was unremarkable. After five months, he developed fever (T >38 °C). At that time, he was receiving tacrolimus, cellcept and prednisolone. His laboratory findings revealed an ALT of 85 IU/L, AST 86 IU/L, Alk-P 730 IU/L, total billirubin of 2.5 and direct billirubin of 0.7 mg/dL. C-reactive protein (CRP) and Epstein-Barr virus viral capsid antigen IgM (EBV VCA IgM) were also positive. Histopathologic examination of the transplanted liver biopsy showed the above photomicrograph. 

**Figure 1:**
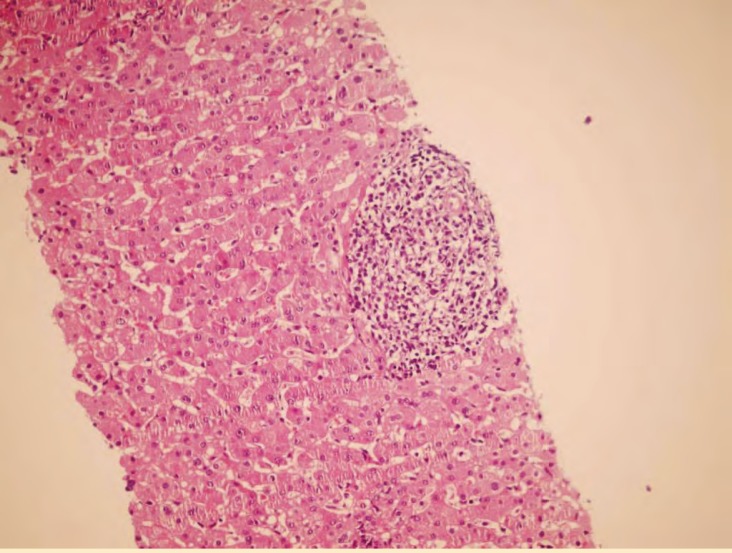
Section from liver needle biopsy shows portal tract infiltration of highly atypical mononuclear cells (H&E, 250×).

**WHAT IS YOUR DIAGNOSIS?**

**DIAGNOSIS:** POST-TRANSPLANT LYMPHOPROLIFERATIVE DISORDER, MONOMORPHIC TYPE, DIFFUSE LARGE B CELL.

Post-transplant lymphoproliferative disorder (PTLD) consists of a heterogeneous group of lymphoproliferative disorders which occurs in about 3% of OLT patients. The incidence of PTLD depends on the age of patient and immunosuppressive therapy used [1]. The current WHO classification is composed of “early lesions,” “polymorphic PTLD,” “monomorphic PTLD” and “Hodgkin’s lymphoma” [2]. PTLD in OLT patients typically presents as a systemic illness, sometimes with involvement of hepatic allograft [3]. It typically occurs as a diffuse infiltration, which mainly involves portal tracts that may be difficult to distinguish from other causes of portal inflammation, including rejection. Immunohistochemical staining for B-cell markers (*e.g.*, CD20) confirms the predominantly B-cell nature of the lymphoid infiltration; which is helpful in differential diagnosis with acute rejection [4]. In rejection, infiltration is mainly composed of T-cells and eosinophils. There is variable involvement of the liver lobules, in some cases with a mononucleosis-like pattern and sinusoidal infiltration. Solid masses may also develop [5]. Our case was biopsied to exclude rejection. The liver needle biopsy section showed infiltration of highly atypical cells which were all positive for CD20 and other B-cell markers, but negative for CD3 and other T-cell markers. Immunohistochemistry for LMP-1 was also positive in some of the atypical cells. After the diagnosis of PTLD and decreasing immunosuppressive therapy, the patient remained febrile and developed cervical lymphadenopathy. The lymph node was excised and showed the same picture of diffuse large B-cell lymphoma. Chemotherapy was started with good response.

PTLD should be considered in all liver transplanted patients with elevated liver enzymes, especially when it is associated with fever.
